# Identification of RAD17 as a candidate cancer predisposition gene in families with histories of pancreatic and breast cancers

**DOI:** 10.1186/s12885-024-12442-z

**Published:** 2024-06-13

**Authors:** Sofie Joris, Philippe Giron, Catharina Olsen, Sara Seneca, Alexander Gheldof, Shula Staessens, Rajendra Bahadur Shahi, Sylvia De Brakeleer, Erik Teugels, Jacques De Grève, Frederik J. Hes

**Affiliations:** 1 Clinical Sciences, Research Group Reproduction and Genetics, Centre for Medical Genetics, Universitair Ziekenhuis Brussel (UZ Brussel), Vrije Universiteit Brussel (VUB), Laarbeeklaan 101, Brussels, 1090 Belgium; 2 The Oncology Research Center, the Laboratory for Medical & Molecular Oncology (LMMO), Faculty of Medicine, Vrije Universiteit Brussel (VUB), Brussels, Belgium

**Keywords:** Pancreatic cancer, Genetic predisposition, Whole exome sequencing, DNA damage repair genes

## Abstract

**Background:**

Among the 10% of pancreatic cancers that occur in a familial context, around a third carry a pathogenic variant in a cancer predisposition gene. Genetic studies of pancreatic cancer predisposition are limited by high mortality rates amongst index patients and other affected family members. The genetic risk for pancreatic cancer is often shared with breast cancer susceptibility genes, most notably *BRCA2, PALB2, ATM* and *BRCA1*. Therefore, we hypothesized that additional shared genetic etiologies might be uncovered by studying families presenting with both breast and pancreatic cancer.

**Methods:**

Focusing on a multigene panel of 276 DNA Damage Repair (DDR) genes, we performed next-generation sequencing in a cohort of 41 families with at least three breast cancer cases and one pancreatic cancer. When the index patient with pancreatic cancer was deceased, close relatives (first or second-degree) affected with breast cancer were tested (39 families).

**Results:**

We identified 27 variants of uncertain significance in DDR genes. A splice site variant (c.1605 + 2T > A) in the *RAD17* gene stood out, as a likely loss of function variant. RAD17 is a checkpoint protein that recruits the MRN (MRE11-RAD50-NBS1) complex to initiate DNA signaling, leading to DNA double-strand break repair.

**Conclusion:**

Within families with breast and pancreatic cancer, we identified *RAD17* as a novel candidate predisposition gene. Further genetic studies are warranted to better understand the potential pathogenic effect of *RAD17* variants and in other DDR genes.

**Supplementary Information:**

The online version contains supplementary material available at 10.1186/s12885-024-12442-z.

## Introduction

Pancreatic cancer (PC) is currently the twelfth most common cancer worldwide, as well as the seventh leading cause of cancer-related death, and its incidence is rising. It generally has a very poor prognosis, with an overall 5-year survival rate of only 10% [1]. The highest incidence and mortality rates are found in Europe, North America and Australia/New Zealand. In Europe, PC is predicted to become the third most common cause of cancer-related death by 2025 [2]. In terms of treatment options, at present surgical resection is the only potentially curative therapy. As PC is asymptomatic in its early stages, advanced non-curable disease at the time of diagnosis is commonly seen [3]. Identifying at-risk individuals is therefore imperative as surveillance increases the probability of early detection of PC.

PC is a multifactorial disease in which environmental factors play a leading role. Known risk factors include exposure to exogenous carcinogens in tobacco and alcohol, diabetes mellitus and chronic inflammation [4, 5]. In addition, as yet unidentified carcinogenic etiologies are highlighted by highly prevalent somatic *KRAS* variants [6, 7]. Despite the sporadic occurrence of most PC, up to 10% occur in a familial context termed Familial Pancreatic Cancer (FPC) [8], which is defined by the presence of at least two first-degree relatives or three relatives with pancreatic cancer. A genetic etiology can be elucidated in a proportion of FPC cases [9], and healthy individuals in an FPC family often have an increased lifetime risk of developing pancreatic cancer [10]. However, an identifiable genetic predisposition is currently found in less than half of all familial PC, accounting for under 5% of all pancreatic cancers. Identified pathogenic variants (PVs) confer variable levels of PC risk, and variants are predominantly found in genes predisposing to breast cancer (*ATM, BRCA1, BRCA2, PALB2*) and colorectal cancer (*MLH1, MSH2, MSH6, PMS2* and *EPCAM*) [10, 11]. In addition, *STK11, CDKNA2* and *TP53* PV carriers also show an elevated risk for PC [12]. These genes share the commonality that they predispose for several cancers. With the exception of the *PRSS* gene [13], which causes hereditary pancreatitis, no other susceptibility gene predisposes exclusively for PC. Analysis of gene panels that include the above mentioned genes has an estimated 10–20% diagnostic yield in PC families [14], which of course means that at least 80% of PC families remain genetically unexplained. In addition, the infrequency of FPC and high short-term lethality of PC further hamper the identification of germline PVs.

Interestingly, a study has shown that 10–20% of pancreatic cancer patients harbor a DDR deficiency in the absence of a detectable BRCA mutation [15]. Since PC has a shared genetic etiology with breast cancer [16], we hypothesized that testing a cohort of combined pancreatic and breast cancer families might reveal additional predisposition genes [17]. In view of the role of many of the above mentioned cancer predisposition genes in DNA repair, in this study we chose to focus on DNA damage response (DDR) genes [18].

During each cell division the DNA replication machinery has a risk of error in response to which cells have developed complex systems to detect and repair these errors. When these mechanisms fail, the DNA damage response is disrupted, allowing damaged cells to survive and in some cases progress to uncontrolled cell proliferation. Dysregulation of DDR also causes genomic instability, a hallmark of cancer [19, 20]. On the other hand, failure of DDR is a weakness that can be targeted therapeutically, as in the case of the PARP inhibitor, Olaparib, an inhibitor that has shown considerable promise in patients with several cancer types harboring germline mutations in *BRCA1/2*, including ovarian, breast and pancreatic cancers [21–23]. Ongoing studies are evaluating PARP inhibitors in patients with tumors exhibiting ‘BRCAness’, a term indicating molecular features shared with BRCA-mutant tumors, which may include a defect in homologous recombination repair and therefore sensitivity to platinum-based agents or PARP inhibitors [24].

In this study we tested a panel of 276 DDR genes in families with both pancreatic and breast cancer, with the goal of identifying novel cancer predisposition genes.

## Methods

Our hospital’s baseline database comprises over 3000 families with familial cancer, accrued between 1994 and 2018. We retrospectively identified families (with at least three breast cancer patients and one pancreatic cancer patient) that had previously been counseled and genetically tested in our familial cancer clinic.

In total, 135 families (4.5%) fulfilled the study’s eligibility criteria. The diagnostic genetic panels previously tested in these families included the *BRCA1, BRCA2* and *CHEK2* genes (up to 2017), and more recently the *ATM, BRCA1, BRCA2, BARD1, BRIP1, CHEK2, MLH1, MSH2, MSH6, PALB2, RAD51C, RAD51D* and *TP53* genes (since 2017). Families found to carry a known cancer predisposition gene (32/135, 23.7%) were excluded. Of the remaining families eligible for the present study (*n* = 103), 62 were excluded due to loss of contact, no interest or other reasons. Finally, 41 families could be included in the current study. The study was approved by the Ethics Committee of the UZ Brussel (BUN 143,201,837,796) and informed consent was obtained from each patient undergoing whole exome sequencing (WES).

**Fig. 1:**
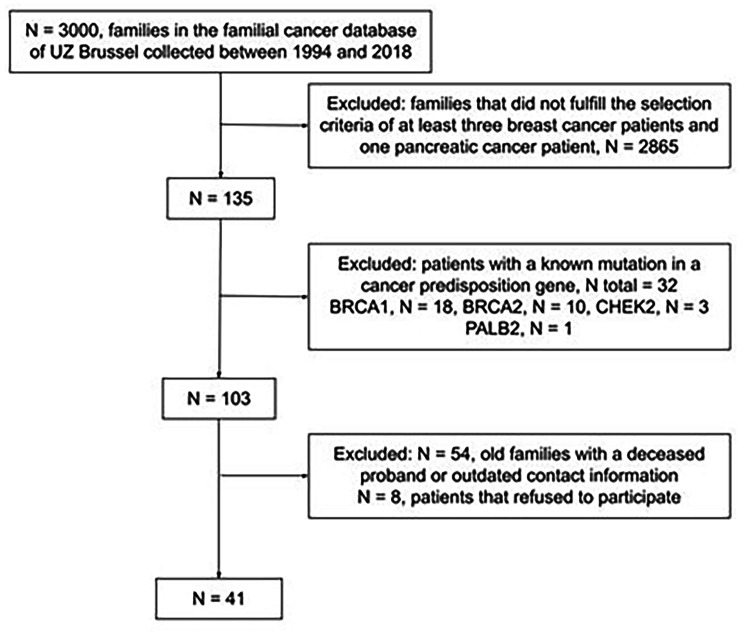
Family selection workflow. Using our selection criteria, 41 families were eligible for our study

### Sequencing

After chemically fragmenting 150ng of genomic DNA extracted from blood, a DNA library was prepared using the KAPA Hyper Plus Prep kit according to the manufacturer’s instructions. This was followed by capture of up to eight DNA libraries using Roche Nimblegen SeqCap EZ Choice XL enrichment probes. The captured fragments were then amplified and sequenced in 2 × 100 bp paired-end mode on an Illumina NovaSeq6000 instrument.

### Data processing

Data analysis was performed using version 3.7 of an (in-house developed) analysis pipeline. In brief, after demultiplexing, the quality of reads was determined with FastQC (v.0.10.0). The reads were then aligned against human reference genome hg19 (ucsc.hg19.fasta) with BWA-mem (v.0.7.10). The aligned reads were sorted and quality control was carried out with samtools v.0.1.19. Duplicate reads were flagged with Picard (v1.97), the reads were further optimized with GATK (v3.3), and then subjected to quality control with Picard (v1.97). The coverage of the final alignment was determined with mosdepth v0.3.1 and ad randomly bounded to 800x with samamba v0.8.0. MLPA data analysis was conducted in Coffalyser.net v04 (MRC Holland), while variant filtration and annotation were conducted in Highlander (16.1).

**Fig. 2:**
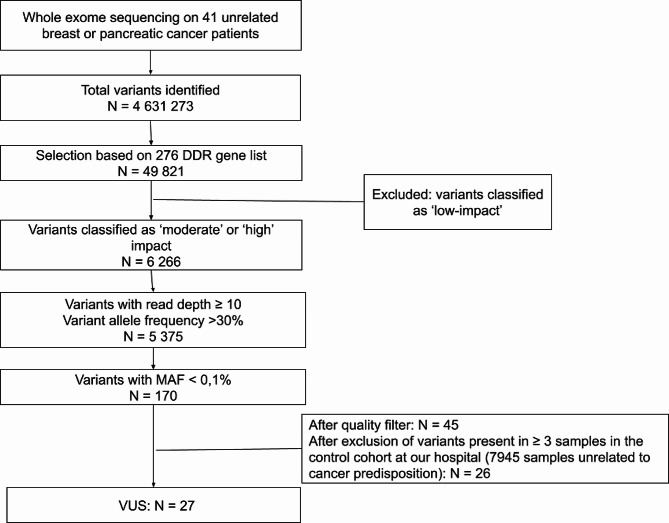
Variant selection workflow

The workflow analysis is illustrated in Fig. 2. First, variants were retained if they were located in genes in the 276 DDR gene list [18] (the genes can be found in supplementary Table 1 of reference 18). The DDR gene list was originally assembled from relevant gene lists, including MSigDB v5.0 [25] (an online catalog of DDR genes from recently published sources [26] as well as information on specific DNA repair pathways or subpathways [27, 28]). In a second step, we retained variants with a moderate to high impact within exons or at splice sites (± 10 bp from the exon-intron border), and low impact variants were excluded. More criteria for retaining included: an absolute read depth > 10x at the variant position, a variant allele ratio higher than 30% and a minor allele frequency (MAF) ≤ 0.1% in population databases. A MAF choice of 0.1% might be considered very strict in some scenarios but was deemed adequate for our goal of identifying a monogenic causative variant. The classification of variants to one of three categories was based on the following: (i) high-impact included nonsense or frameshift mutations predicted to change gross protein structure or mutations predicted to affect splicesites; (ii) moderate impact were the non-synonymous variants; (iii) the low-impact category consisted of synonymous variations in coding regions and variants in non-coding regions (upstream, downstream, intergenic and UTR regions). PubMed, ACMG guidelines and REVEL, an ensemble method for predicting the pathogenicity of missense variants based on a combination of scores from 13 individual tools [29], were consulted concerning variant classification.

DDR = DNA damage repair, VUS = variant of uncertain significance, MAF = minor allele frequency.

## Results

Using WES, we identified 4,631,273 genetic variants in the 41 persons tested. Based on a reference list of 276 DDR genes previously associated with cancer, this number was narrowed to 49,821 variants. After additional filtering (Fig. 1), we identified 27 variants of uncertain significance (VUS). A splice site variant (c.1605 + 2T > A) in the RAD17 gene stood out, as a likely loss of function variant. All variants were classified based on the literature consulted via PubMed as well as the ACMG guidelines (see Table 1).

**Table 1:** Identified variants ranked by GnomAD frequency. The first column contains the HUGO gene acronym. The next column shows the NM accession number, which links to the mRNA record in the NLM NCBI nucleotide database. Column 3 shows cDNA variant annotation and column 4 the predicted protein variant annotation. The last four columns consist of guidelines and tools used to categorize the variants (ACMG (American College of Medical Genetics) guidelines [30], Clinvar [31] and gnomAD v.2.1.1 [32] (Genome Aggregation Database)). Abbreviations: ACMG: The American College of medical genetics and genomics: Alt: alteration: AMP: the association of molecular pathology: LP: likely pathogenic: NF: non-Finish: NR: not reported: P: pathogenic: PIN: patient identification number: ref freq: reference frequency: VUS: variant of uncertain significance

PIN	Gene	transcript	cDNA	protein level	ACMG	Clinvar	GnomAD (all)	GnomAD (NF.)
						database	(alt/ref freq)	(alt/ref freq)
21	RAD17	NM_133339.1	c.1605 + 2T > A	p.?	CL 3	NR.	0	0
38	BLM	NM_000057	c.3538G > A	p.Val1180Ile	CL 3	VUS	0	0
13	FANCD2	NM_033084	c.1010G > T	p.Ser337Ile	CL 3	NR	0	0
28	MLH3	NM_001040108	c.2257T > G	p.Ser753Ala	CL 3	NR	0	0
30	PNKP	NM_007254	c.1436T > C	p.Met479Thr	CL 3	NR	0	0
30	SLX1B	NM_024044	c.620G > A	p.Cys207Tyr	CL 3	NR	0	0
13	TOP3A	NM_004618	c.1747G > T	p.Asp583Tyr	CL 3	NR	0	0
37	POLH	NM_006502	c.34G > A	p.Val12Met	CL 3	NR	0.00079524% (1)	0% (0)
38	PNKP	NM_007254	c.1442G > T	p.Gly481Val	CL 3	NR	0.0007956% (1)	0.0017593% (1)
15	RAD17	NM_133339.1	c.1337G > A	p.Gly446Glu	CL 3	NR	0.00082154% (1)	0.001797% (1)
33	HLTF	NM_003071	c.2957G > C	p.Gly986Ala	CL 3	NR	0.0014584% (2)	0.0031722% (2)
19	ERCC4	NM_005236	c.1603G > C	p.Asp535His	CL 3	NR	0.0015912% (2)	0
11	POLG	NM_001126131	c.3487 A > G	p.Met1163Val	CL 3	VUS	0.0015912% (2)	0.0017587% (1)
24	ERCC3	NM_000122	c.1468G > A	p.Glu490Lys	CL 3	NR	0.0015931% (2)	0.0017615% (1)
34	DNA2	NM_001080449	c.3322T > A	p.Cys1108Ser	CL 3	NR	0.0016048% (2)	0% (0)
13	MBD4	NM_003925	c.494 C > G	p.Ser165Cys	CL 3	NR	0.0031846% (4)	0.0070393% (4)
20	POLA1	NM_016937	c.3925 C > T	p.Arg1309Cys	CL 3	NR	0.0046992% (3)	0.0071003% (2)
18	SWSAP1	NM_175871	c.641 C > G	p.Ala214Gly	CL 3	NR	0.0056994% (8)	0
30	PER1	NM_002616	c.1988 C > G	p.Ser663Cys	CL 3	NR	0.010376% (13)	0.010595% (6)
30	PER1	NM_002616	c.1996G > T	p.Asp666Tyr	CL 3	NR	0.010612% (6)	0.01039% (13)
12	RECQL4	NM_004260	c.2086 C > T	p.Arg696Cys	CL 3	VUS	0.011712% (11)	0.020882% (8)
38	FANCD2	NM_033084	c.1757 C > T	p.Ala586Val	CL 3	VUS	0.014143% (20)	0.021676% (14)
36	ERCC2	NM_000400	c.2260G > C	p.Glu754Gln	CL 3	VUS	0.021976% (31)	0.048178% (31)
22	APLF	NM_173545	c.1142 A > G	p.Tyr381Cys	CL 3	NR	0.024336% (33)	0.047007% (29)
22	FANCM	NM_020937	c.538 A > G	p.Ile180Val	CL 3	VUS	0.024759% (35)	0.044914% (29)
28	USP19	NM_001199160	c.10G > A	p.Gly4Arg	CL 3	NR	0.027269% (38)	0.04835% (31)
7	TP53BP1	NM_001141980	c.2226 A > T	p.Glu742Asp	CL 3	NR	0.072362% (99)	0.087199% (55)

The c.1605 + 2T > A, p.? variant in *RAD17* (NM_133339.1) is a nucleotide substitution in the canonical splice donor site of exon 13. Splice site predictions tools such as SpliceSiteFinder-like, MaxEntScan, NNSPLICE and Genesplicer (a score of -100% in all cases) indicate a very strong likelihood of leading to aberrant splicing. Since we are the first to associate *RAD17* with this phenotype and due to the lack of further segregation or functional studies, we had to classify the variant as a class 3 according to the ACMG guidelines.

**Fig. 3:**
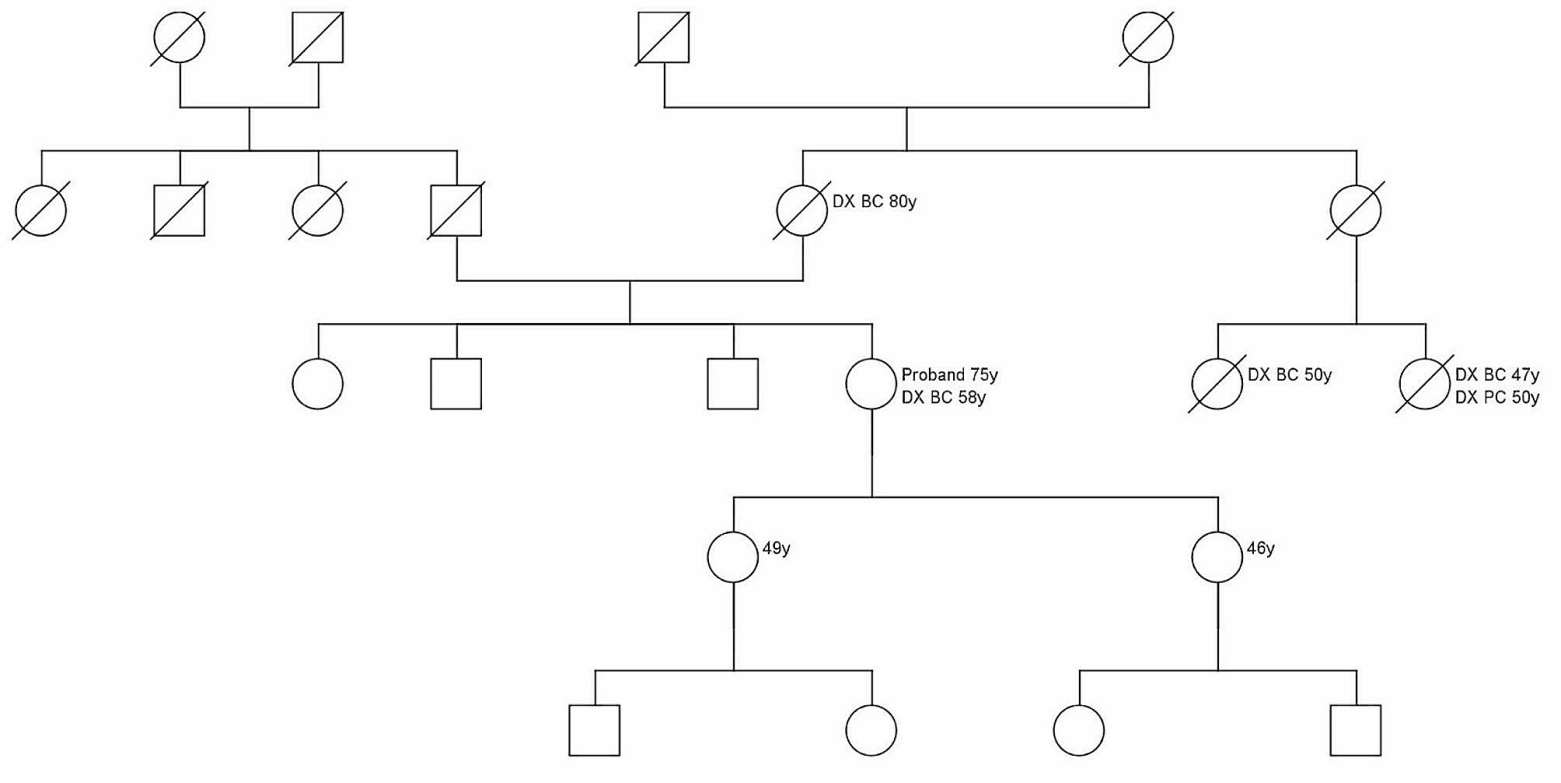
Pedigree of the *RAD17*, c.1605 + 2T > A variant, with ages at diagnosis (Dx) of cancer. BC: breast cancer: PC: pancreatic cancer

The variant was found in a small high-risk family (pedigree is shown in Fig. 3) in which the proband was diagnosed with breast at age 58. Her mother had breast cancer at age 80. In addition, two maternal cousins were diagnosed with cancer, one with breast cancer at age 50 and one with breast and pancreatic cancer at the ages of 47 and 50, respectively. Their mother, the maternal aunt of the proband, died at a young age while giving birth to her second child. As all patients with exception of the proband were deceased, we were unable to perform segregation analysis in this family. There was also no tumor tissue available for segregation analysis or loss of heterozygosity (LOH) studies.

Of the other 26 identified variants of uncertain significance, two variants, NM_024044 (*SLX1B*): c.620G > A, p.Cys207Tyr, and NM_000122 (*ERCC3*): c.1468G > A, p. Glu490Lys, are of particular interest, since they showed a REVEL (Rare Exome Variant Ensemble Learner) score of > 0.7.

The *SLX1B* c.620G > A variant impacts a genetically well-conserved amino acid and is not present in general population databases (GnomAD v2.1.1). Interestingly, this gene encodes a protein that plays an important role in maintaining genome stability.

**Fig. 4:**
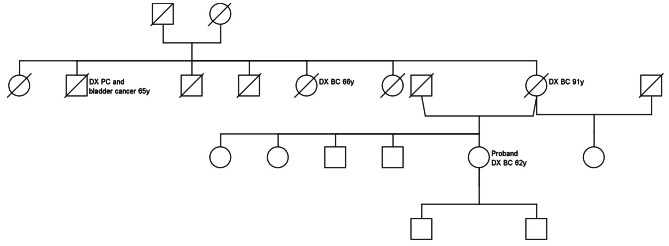
Pedigree carrying the *SLX1B* c.620G > A variant, with ages of cancer diagnosis (Dx). BC: breast cancer: PC: pancreatic cancer

In the family with this variant (pedigree depicted in Fig. 4) the proband had breast cancer at age 62 years, a maternal uncle had pancreatic cancer at age 65, while her mother and a maternal aunt had breast cancer at ages 92 and 68, respectively. The maternal aunt was not available for segregation analysis.

**Fig. 5:**
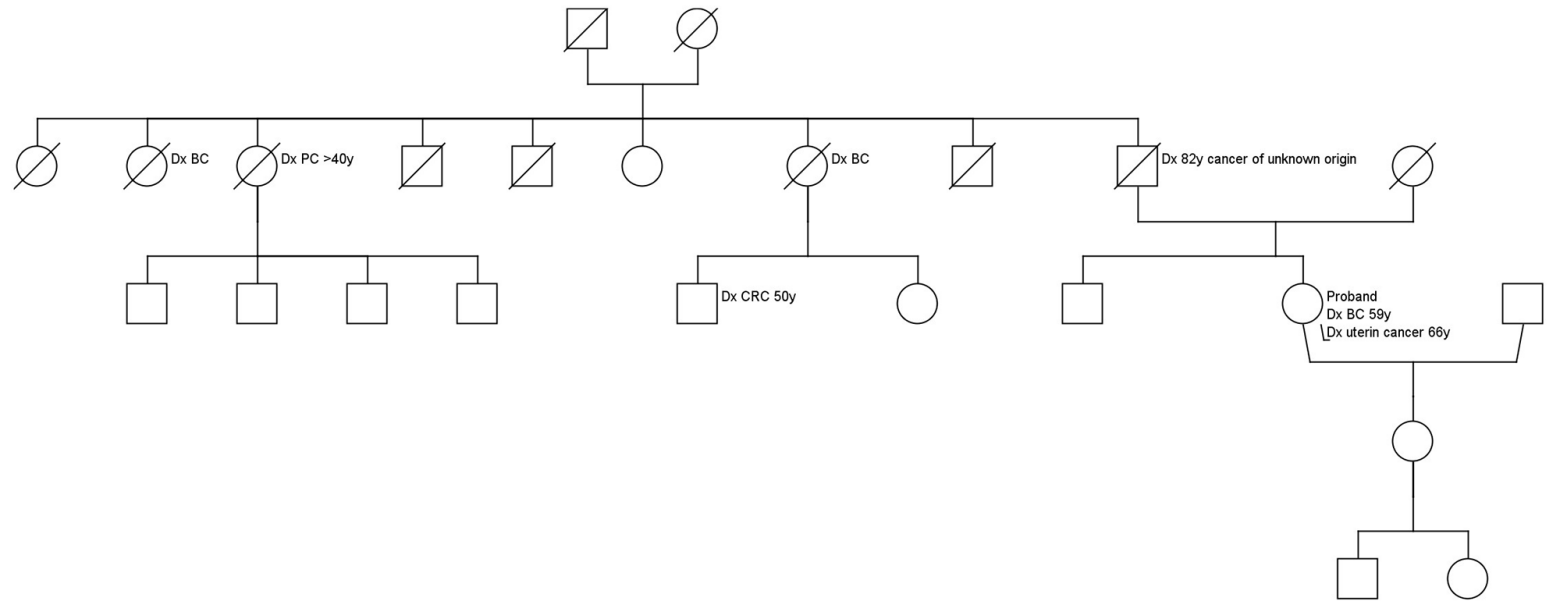
Pedigree of the *ERCC3* c.1468G > A variant, with ages of cancer diagnosis (Dx). BC: breast cancer: CRC: colorectal cancer: PC: pancreatic cancer

The second VUS, c.1468G > A, p.Glu490Lys, was found in the excision repair 3 (*ERCC3*) gene (NM_000122). Excision repair genes are crucial members of the nucleotide excision repair (NER) pathway. The family (pedigree shown in Fig. 5) comprised a proband with breast cancer at age 59 years, as well as uterine cancer at age 66. Her father had a cancer of uncertain origin at the age of 82, a paternal aunt had pancreatic cancer (age unknown) and two paternal aunts were diagnosed with breast cancer (ages unknown).

## Discussion

In this study we performed whole exome sequencing together with targeted cancer gene panel analysis of 276 DDR genes in 41 families with PC, either in index patients or close relatives with breast cancer. A similar strategy of sequencing breast cancer-affected relatives of deceased PC patients has been adopted in other studies, such as a recent WGS (whole genome sequencing) study that identified rare genetic mutations in cancer-related genes in first degree relatives of PC patients [17].

Our motivation to focus on DDR genes was partly due to the fact that dysregulation of DDR causes genomic instability, an important hallmark of cancer, as well as the potential benefit regarding targeted therapy of tumors with novel alterations in DDR pathways. Non-BRCA homologous recombination repair (HRR) variants are relatively common (7%), as confirmed in a recent NGS study in which germline DNA of PC patients was analyzed using a multigene panel of 21 HRR genes, and it has been suggested that carriers of HRR variants may benefit from treatment with PARP inhibitors [33].

The most important finding of this study was the c.1605 + 2T > A mutation in *RAD17*, as germline mutations in *RAD17* have not been previously identified in hereditary cancer syndromes. By contrast, somatic mutations in *RAD17* have been found in several types of cancer [34] including PC (7%), together with features that support a tumor suppressor role such as LOH and biallelic loss [35].

*RAD17* plays an essential role in recruiting the MRN complex (MRE11-RAD50-NBS1), which is fundamental to the detection of DNA double-strand breaks and the initiation of DNA damage signaling [36]. A reduction or loss of RAD17 protein may therefore lead to an increased risk of cancer and genomic instability.

Interestingly, exon 13 encodes the C-terminal alpha-helical domain of *RAD17* [37]. This amino acid sequence contains a conserved motif, the PCNA-interacting protein (PIP) box, which mediates the interaction of RAD17 with PCNA (proliferating cell nuclear antigen), a protein involved in DNA replication and repair. This interaction is critical for the activation of the replication checkpoint, which ensures that DNA replication and error correction proceed smoothly. Additionally, the interaction between RAD17 and PCNA is essential for the adequate assembly and loading of the RAD9-RAD1-HUS1 (9-1-1) complex, which acts as a DNA damage sensor and activates checkpoint pathways in the response to DNA damage [38, 39]. In addition, impairment of RAD17 function through miR-506-3p in vitro has been found to induce a “BRCAness” phenotype, as they show reduced DNA damage responses and induced platinum sensitization [40].

The *RAD17* c.1605 + 2T > A variant is a nucleotide substitution in the canonical splice donor site of exon 13, leading to a predicted loss of the splice donor site of exon 13, which is expected to lead to exon skipping of a well preserved region in the protein. Unfortunately, we lack the possibility to further investigate the biological impact of this splice site variant in patient derived samples. The proband could not be motivated to collaborate for the necessary additional blood sampling needed for RNA sequencing. However, the given variant is predicted to result in the in-frame deletion of amino acids 486–535. This region forms part of a domain required for the interaction with MCM7, which in turn is mandatory for replication checkpoint signaling [41]. In this article, depletion of either hRad17 or hMCM7 with small-interfering RNA suppressed ultraviolet (UV) light- or aphidicolin-induced hChk1 phosphorylation, and abolished UV-induced S-phase checkpoint activation. We postulate that the RAD17 splice variant leads to a loss of function of a protein involved in the DNA damage repair, hence functioning a tumor suppressor gene. Further, the variant is absent from controls (gnomAD v.2.1.1). Taken together, the combination of a loss of function variant and its absence from controls, qualifies *RAD17* as a cancer predisposition gene. However, a direct link with pancreatic cancer predisposition remains uncertain, due to incomplete segregation, as the aunt with pancreatic cancer was already deceased. Therefore, further germline testing of pancreatic cancer patients for DDR genes, including *RAD17*, may further establish their role in cancer predisposition.

Concerning PARP inhibitors, current policy for most cancers allows reimbursement only when a *BRCA1* or *BRCA2* alteration has been confirmed. However, in some countries reimbursement also covers treatment of ovarian cancer when HRD (homologous recombination deficiency) is present in cancer cells. Ongoing studies are exploring the PARP inhibitor response in patients with ‘BRCAness’, a term that refers to tumors that share molecular features with BRCA-mutant tumors. Given the role of DNA damage checkpoints in homologous recombination, tumor sensitivity to PARP inhibition and platinum-based chemotherapy should be investigated in relation to germline mutations in *RAD17* [21–23, 42]. Preclinical data also indicate that DDR defects increase sensitivity to gemcitabine. Therefore, *RAD17* mutations are an interesting and potentially actionable addition to previously identified genes in the DNA damage response (DDR) pathway in pancreatic cancer and breast cancer [42–45].

In addition to a mutation in *RAD17*, we also found twenty-six unique variants of uncertain significance (Table 1) of which two, p.Cys207Tyr in *SLX1B* and p. Glu490Lys in *ERCC3*, had a REVEL score > 0.7 and thus may be of interest. *SLX1B* encodes the catalytic subunit of the SLX1-SLX4 structure-specific endonuclease, which can resolve DNA secondary structures formed during repair and recombination processes [46]. Read-through transcription between this gene and the downstream SULT1A4 (sulfotransferase family, cytosolic, 1 A, phenol-preferring, member 4) gene [47] produces a SLX1B-SULT1A4 fusion protein that is important in DNA repair.

*ERCC3* is a crucial member of the NER pathway, deficiencies of which result in a heterogeneous group of disorders ranging from UV-sensitive syndrome to cancer-prone xeroderma pigmentosum, as well as the neurodevelopmental/progeroid conditions Trichothiodystrophy, Cockayne syndrome and Cerebro-oculo-facio-skeletal-syndrome [16]. However, in the literature *ERCC2* and *ERCC3* have no dominant link with cancer, whereas polymorphisms in ERCC4 have been linked to cancer predisposition [17].

## Conclusion

We identified *RAD17* as a novel candidate cancer predisposition gene in a breast and pancreatic cancer family. A genotype-phenotype correlation is to be further established. Further molecular genetic analyses are required to validate the effect of the c.1605 + 2T > A variant, and warrant further exploration of *RAD17* and DDR genes in additional familial pancreatic and breast cancer cohorts.

### Electronic supplementary material

Below is the link to the electronic supplementary material.

Supplementary Material 1

## Data Availability

All the relevant data are included in this article. The sequencing data after filtering are available at https://www.dropbox.com/scl/fi/bjpqbhgdqui46if6wbu9z/all-variants-additional-file.xlsx?rlkey=swi0p2v3qqa15hm265pm80tll&dl=0. Due to legal concerns with regard to the DPA (data processing agreement), raised by the legal department of the UZ Brussel and GDPR (General Data Protection Regulation) in Belgium we were unable to upload the raw sequencing data at EGA (European Genome-Phenome Archive). All Belgian universities are currently working together to develop a national registry so that it can be used in the future to store and share research data in case of a publication.
